# Expectations and Experiences of Peer Mentors in Supporting People With Osteoarthritis and Socioeconomic Disadvantage: A Multi‐Method Exploration of Challenges and Lessons Learned

**DOI:** 10.1111/hex.70819

**Published:** 2026-08-16

**Authors:** Elizabeth Lavender, Samantha Mason, Anna M. Anderson, Linda Eckersley, Susan Barry, Gretl A. McHugh

**Affiliations:** ^1^ School of Healthcare University of Leeds Leeds UK; ^2^ Leeds Institute of Health Sciences University of Leeds Leeds UK; ^3^ Leeds Institute of Rheumatic and Musculoskeletal Medicine University of Leeds Leeds UK; ^4^ NIHR HealthTech Research Centre in Accelerated Surgical Care Leeds UK

**Keywords:** long‐term conditions, osteoarthritis, peer mentorship, self‐management, socioeconomic disadvantage

## Abstract

**Introduction:**

Osteoarthritis and other multiple long‐term conditions disproportionately affect people experiencing socioeconomic disadvantage, leading to complex and intersecting health and wellbeing needs. Peer mentorship can support self‐management and complement condition specific care. Delivering effective peer mentorship remains challenging.

The overall study aim was to develop and assess feasibility and perceived usefulness of a remotely delivered osteoarthritis peer mentorship intervention. This paper explores peer mentors’ experiences of delivering the peer mentorship programme, their preparedness for the role and the challenges they faced.

**Methods:**

Fourteen volunteers were trained as peer mentors to deliver the peer mentorship programme to 30 mentees. Mentors completed training evaluations, summaries of mentorship sessions and participated in a semi‐structured qualitative interview. Data sources were combined to form a narrative of the peer mentors’ perceptions and experiences, pre and post intervention delivery.

**Results:**

Twelve peer mentors completed training evaluations, sessions summaries and were interviewed. Three key themes were developed: aligning expectations of the peer mentoring role with mentoring experience; preparedness for peer mentoring; and peer mentorship delivery challenges.

**Conclusions:**

Peer mentorship is a potentially valuable approach to supporting people to self‐manage long‐term conditions such as osteoarthritis. Findings offer practical insights for designing peer mentorship programmes that align with mentors’ expectations, provide adequate preparation and support, and address challenges to effective delivery.

**Patient and Public Contribution:**

Public contributors with hip and knee OA and experience of socioeconomic disadvantage helped develop the programme, test remote delivery and recruit mentees. They contributed to peer mentor training and supported peer mentors to deliver the programme.

## 1 Introduction

It is estimated that over 10 million people in the UK have osteoarthritis (OA) [1]. This long‐term condition most commonly affects hip, knee and hand joints. Symptoms of OA include chronic pain, reduced mobility and fatigue, often negatively impacting people's ability to work and quality of life [2]. The prevalence of OA increases with age and disproportionately affects people experiencing socioeconomic disadvantage [3, 4, 5]. People living with OA are 2.5 times more likely to have two or more additional long‐term conditions such as cardiovascular disease and diabetes than people without OA [2]. Around 1 in 5 people with OA experience symptoms of depression or anxiety [1, 3]. The prevalence of adults living with multiple long‐term conditions (MLTCs) is greatest in areas of socioeconomic deprivation [6, 7] resulting in a higher proportion of people with complex intersecting health and wellbeing needs in deprived areas. These populations also face multiple barriers to accessing the health and care support they need [8, 9].

Our ageing population and rising prevalence of MLTCs has created an untenable strain on health and social care services [10, 12]. The NHS 10 Year Plan [13] stipulates a move towards preventative care, and to empower individuals to become accountable for their health. Supportive self‐management strategies that educate, encourage and enable people to take control of their condition(s) may play an important role in long‐term condition management, but need to be specifically tailored to be effective for people experiencing socioeconomic disadvantage [6, 9].

Peer support models such as peer mentorship are encouraged for self‐management of long‐term conditions [14, 15, 16, 17], complementing condition specific care and addressing wider needs of patients. However, there are challenges to providing effective peer support due to the varied, informal and often unsupervised nature of the support, and strong reliance on positive interpersonal connections [9, 18, 19].

Peer mentorship is a form of guided peer support for self‐management involving education, skills development, coping strategies, and other helpful activities such as goal setting, activity pacing and signposting to wider support [15, 18, 20]. Peer mentors are people with the same condition as the patient/mentee, who show evidence of positively managing the condition and willingness to share their knowledge and experience to benefit others. Peer mentorship involves complex interpersonal interactions and understanding of wider health and social contexts. To be effective, peer mentors must work to build rapport with their mentees and encourage a sense of empowerment. In addition to sharing lived experience, peer mentors need to have appropriate knowledge and skills to deliver a self‐management programme [21, 22]. Provision of peer mentorship is therefore subject to wide variation. Peer mentors need to know what is expected of them in the role and be adequately prepared and supported to support others.

Our study team previously developed, and feasibility tested an in‐person peer mentorship OA support intervention and training programme [23]. Our study concluded that the intervention appeared feasible and acceptable to both mentees and mentors. To increase potential to reach people facing health inequities, we adapted the intervention for remote delivery to people with OA experiencing socioeconomic disadvantage as part of the ‘Remote Osteoarthritis Peer Mentorship for Socioeconomically Underserved People (RaMIgO) study’. To our knowledge, only one other study has engaged people with hip/knee OA to develop a peer mentorship programme for people with OA [24] and none have specifically engaged people experiencing socioeconomic disadvantage.

The overall aim of this study was to develop and assess the feasibility and perceived usefulness of a remotely delivered osteoarthritis peer mentorship intervention [25, 26, 27]. People with lived experience were trained to become peer mentors and deliver the OA self‐management programme.

Given that peer support plays a significant role in effective self‐management of OA and other long‐term conditions, and peer mentors are central to this intervention, we wanted to explore their experiences of delivering the self‐management programme. This paper focuses on peer mentors’ expectations and experiences of their role, their preparedness to deliver the self‐management programme, the challenges they faced and lessons learned.

## 2 Materials and Methods

### 2.1 RaMIgO Study Overview

The main study was a mixed‐methods feasibility study comprised of three phases. In Phase 1 (intervention development) we adapted our previously developed OA intervention for remote delivery to people experiencing socioeconomic disadvantage [26]. Public contributors/PPI participated in workshops and practice runs of the intervention delivery. Phase 2 (intervention set‐up) involved recruiting volunteer peer mentors and training them to deliver the intervention. Phase 3 (intervention evaluation) was a mixed‐methods process evaluation involving trained peer mentors delivering the intervention to people with OA, and investigating the acceptability, appropriateness, feasibility and fidelity of the intervention [27]. Wider implementation was explored through Stakeholder Discussion Forums.

### 2.2 Intervention Overview

The RaMIgO intervention is an osteoarthritis self‐management programme, including resources and trained volunteers (peer mentors) with lived experience of hip/knee OA and socioeconomic disadvantage [25]. The 6‐week programme was designed to be delivered remotely via videoconferencing (e.g. Zoom or WhatsApp) or telephone to study participants (mentees) one‐to‐one or in small groups. To be eligible to participate, peer mentors and mentees had to: be over 18 years and living with osteoarthritis of the hip or knee (or both); feel disadvantaged due to financial, educational or social circumstances; and able to dedicate a minimum of 1 hour/week for 6‐8 weeks.

All peer mentors and mentees provided informed consent prior to participating. Reporting of this study is guided by COREQ guidelines [28] and a completed checklist is provided (Supporting File 1).

### 2.3 Recruitment of Peer Mentors

Peer mentors were recruited through national and local community agencies via posters, news articles, social media advertising and word of mouth. Recruitment took place over 5 months from August 2023 to January 2024. Interested volunteers were screened for eligibility, provided with a role description (Supporting File 2) and invited to attend an informal online recruitment interview with a member of the study team.

Interviews focused on the volunteer's suitability and motivation for peer mentoring, and expectations of their involvement. Suitability criteria included communication and listening skills, peer support experience, confidence with remote connection and personal characteristics including a desire to help others. Suitable applicants were invited to attend a 2‐day training course.

Thirty‐five people expressed interest in the peer mentor role and twenty‐six were screened. After screening for eligibility and following withdrawals from the study, seventeen volunteers were invited to attend the peer mentor training, with fourteen attending. Following training, thirteen volunteers underwent Disclosure and Barring Service (DBS) checks and became peer mentors.

### 2.4 Peer Mentor Training

Trainees attended a remotely delivered 2‐day training course (January‐February 2024). Training was delivered via videoconferencing in two groups with 6‐8 trainees in each. The course was developed in an earlier phase of the study [25] and delivered by study team members; a physiotherapist specialising in activity pacing and pain management; and Patient and Public Involvement (PPI) Group members with experience of peer mentoring from a previous study [29]. Table 1 provides an overview of peer mentorship training.

**Table 1:** Overview of peer mentorship training.

Topic	Contents
Day One	
Study overview	Introduction to study and study team Peer mentorship – what it is and its place in the study Potential study outcomes
Being a peer mentor	Understanding the peer mentor role and expectations Time commitment and involvement with participants Support provided by study team
Knowledge sharing – OA and peer mentoring	OA key facts (group activity) Self‐management Goal setting
Developing skills	Introduction to resource pack and handouts Overview of core and optional topics Goal setting scenarios (group activity) Exercises (group activity)
Knowledge and skills	Pacing – what it is and why it is important How to implement activity pacing into daily life Understanding pain Persistent pain cycle Breaking the pain cycle and pain management
Healthy eating	Introducing the Eatwell Guide (culturally specific options) What is ‘healthy’ eating
Day Two	
Developing skills	Effective communication Communication styles Developing communication skills (group activity) Peer mentor and mentee relationships Professional boundaries Boundary scenarios (group activity) Peer mentoring in practice and role‐playing scenarios (PPI and group activity)
Knowledge and skills	Practicalities of being a peer mentor (PPI and group activity) Connecting digitally with the mentee Recording session summaries (PPI and group activity) Confidentiality Safeguarding Disclosures

Training was designed to build knowledge about self‐managing OA and develop self‐management and mentoring skills. Training covered topics related to OA, the self‐management programme and peer mentoring, and consisted of educational components, knowledge sharing, development of skills and familiarisation with programme content. To help engagement with remote delivery, training was divided into short sections interspersed with regular breaks, and involved a range of presentations and interactive activities, including practice sessions with experienced past peer mentors.

Peer mentors were given printed and electronic versions of the resource pack and participant handouts prior to training. These were used throughout training and included information and guidance on delivering the self‐management programme. The programme included six core topics, all of which peer mentors were expected to cover; and six optional topics which could be covered if relevant to mentees. Table 2 provides an overview of the peer mentorship programme.

**Table 2:** Overview of peer mentorship programme.

Topic name	Aim
Core topic 1: Self‐managing OA	Provide an understanding of OA and self‐management tools to help mentee to manage their symptoms better
Core topic 2: Setting realistic goals	Provide support to mentee to set and review simple weekly goals that are meaningful to them
Core topic 3: Getting active, staying active	Encourage being physically active as a key part of OA self‐management and as a way to improve overall health and wellbeing
Core topic 4: Eating well, feeling well	Emphasise the multiple benefits of healthy eating, including helping OA pain through weight management
Core topic 5: Activity pacing in practice	Provide an understanding of activity pacing; boom‐bust cycle where people over‐do activities on days where their symptoms are less severe, leading to a flare‐up of their symptoms and needing a period of rest
Core topic 6: Managing pain	Introduce self‐management strategies and tools to help manage pain better
Optional topic 1: Getting motivated	Support mentees to find solutions that will help to motivate them to try self‐management
Optional topic 2: Relaxing and sleeping	Introduce techniques that may help a mentee to relax and sleep better
Optional topic 3: Communicating well	Improve communication in a way that can help relationships with family, friends and health professionals. Help the mentee to communicate assertively with healthcare professionals and better advocate for themselves
Optional topic 4: Getting connected	Encourage the mentee to make other connections that will help their wellbeing and plan for when the peer mentorship ends
Optional topic 5: Having a joint replaced	Help the mentee to prepare for a joint replacement and for recovery afterwards
Optional topic 6: Getting support for work	Inform the mentee of support options that may help if the mentee's OA symptoms are affecting their ability to work

The peer mentors were matched with recruited study participants/mentees. Mentees were 30 people with hip and knee OA self‐identifying as experiencing socioeconomic disadvantage. They varied in age, gender, ethnicity, site of OA, length of time with OA, digital capability and UK geographical location. Mentees were recruited via GP practices serving areas of high levels of socioeconomic disadvantage, a Community Healthcare NHS Trust; national and local third sector organisations; a specialist community health recruitment agency; PPI community networks; and social media. Mentee recruitment and participation is further reported elsewhere [25, 27].

Mentor‐mentee matching was predominantly based on availability of mentors and mentees, personal characteristics (e.g. self‐assured, adaptable, empathetic), preference for mentoring via telephone or videoconferencing, and for group or 1‐1 mentoring. In some cases matching was based on mentoring experience, ethnicity and gender. For example, more experienced mentors were invited to run small groups (with 2 or 3 mentees).

Individual peer mentors were matched with between 1 and 5 mentees and met individually with one mentee or with a pair of mentees in small groups. The content of the peer mentorship programme was intended to be the same for individual and small group sessions.

Peer mentors in this study were predominantly White British (77%) female (77%) aged between 32 and 72 (average 59 Years). Most (85%) were part‐ or full‐time employed or actively volunteering. Table 3 provides a summary of mentor demographics and mentoring activity.

**Table 3:** Peer mentor demographics and mentoring activity.

Participant characteristic	*n* (%)
Gender	
Male	3 (23.1)
Female	10 (76.9)
Age (years)	
Range	32–72
Median	59
Ethnicity	
White British	10 (76.9)
White European	1 (7.7)
Black Caribbean	1 (7.7)
British Pakistani	1 (7.7)
Site of OA	
One knee	1 (7.7)
One hip	0 (0)
Both knees	4 (30.8)
Both hips	5 (38.5)
One hip and both knees	2 (15.4)
One knee and both hips	1 (7.7)
Length of time with OA	
< 5 years	3 (23.1)
5‐10 years	2 (15.4)
10‐15 years	4 (30.8)
15‐20 years	2 (15.4)
20‐30 years	1 (7.7)
> 30 years	1 (7.7)
Employment status	
Employed full‐time	1 (7.7)
Employed part‐time	4 (30.8)
Unemployed non‐volunteer	1 (7.7)
Unemployed volunteer	3 (23.1)
Retired non‐volunteer	1 (7.7)
Retired volunteer	3 (23.1)
Number of mentees supported	
1	4 (30.8)
2	4 (30.8)
3	3 (23.1)
4	1 (7.7)
5	1 (7.7)

### 2.5 Programme Delivery

Mentorship sessions were set up by the study team or the peer mentor. Prior to session one, mentees were provided with programme resources including educational handouts, exercise sheets and information booklets from Arthritis UK (previously Versus Arthritis). A member of the study team attended part of the first session to introduce peer mentors and mentees and answer questions about the study. Subsequent sessions were frequently arranged, but not attended, by a study team member.

Although led by the mentor, sessions were intended to be collaborative and tailored to focus on mentees’ individual needs. Peer mentors offered support with self‐management through information sharing, goal setting, skill development and confidence building, motivation, and social support.

Support with scheduling and setting up sessions, structuring sessions and overcoming challenges was provided by the study team and two PPI members/previous peer mentors. Mentors were offered a ‘thank you payment’ of twenty pounds for each mentoring session delivered.

**Figure 1:**
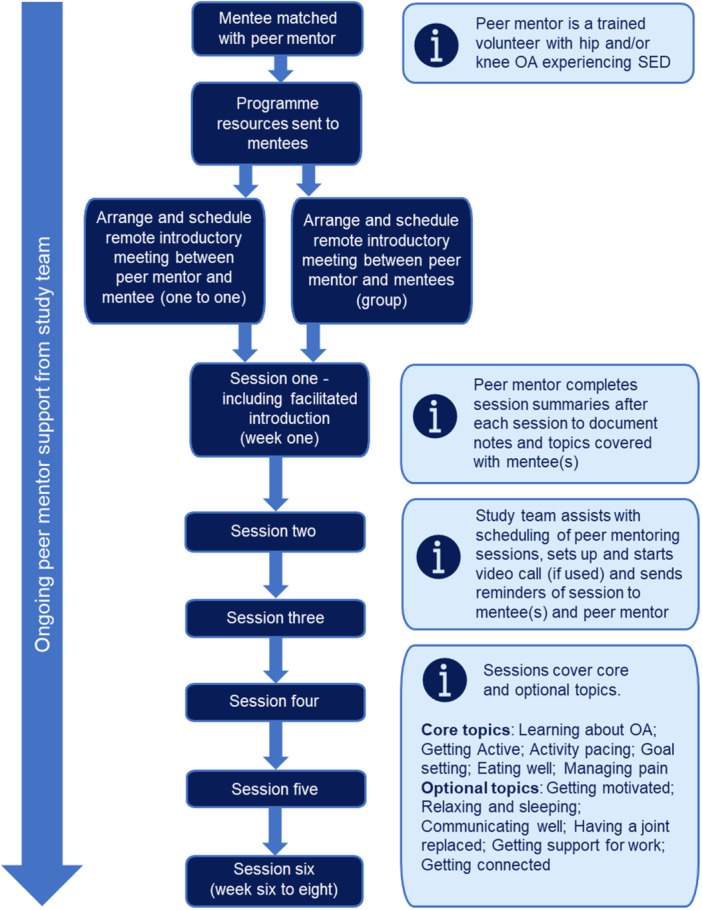
Intervention delivery. OA, osteoarthritis, SED, socioeconomic disadvantage. Additional information on the stages of delivery is provided in the boxes marked with the ‘i’ icon. An image description of the figure is available in Supporting File 3.

Figure 1 provides a detailed overview of the intervention delivery process (Supporting File 3: provides Figure 1 image description).

### 2.6 Patient and Public Contribution

Each phase of this study benefitted from close involvement of eight PPI members, people with lived experience of OA and socioeconomic disadvantage, who contributed to the intervention design and development and helped with recruitment. PPI members helped adapt the peer mentorship programme and test remote delivery. Four PPI members directly contributed to the peer mentor training. In addition, two peer mentors from the previous study supported this study's mentors via email exchanges. One of the previous peer mentors also co‐delivered two mentoring sessions with two peer mentors who needed additional support or lacked the lived experience of having a joint replacement due to OA.

### 2.7 Data Collection

Three sets of data were collected from peer mentors.

#### 2.7.1 Training Evaluation

Immediately following training, participants were asked to evaluate their training experience, including aspects they found more/less engaging, and rate their confidence to deliver the programme by completing an anonymous online or paper evaluation form. The training evaluation was an adaptation of a form used in our previous study [23] developed by the study team and PPI members. It comprised 5‐point Likert scale questions and open text options. We did not test for validity and reliability of training evaluations as we were not measuring psychological constructs relevant to the interview data. We were simply quantifying experiences and recording suggestions for improvement.

#### 2.7.2 Peer Mentor Qualitative Interview

At the end of their mentoring period, all peer mentors were invited (by phone or email) to participate in a single semi‐structured qualitative interview. The aims of the interview were to: understand motivations for wanting to become a peer mentor; explore perceptions of training and support including how well equipped for peer mentoring they felt; explore experiences of delivering the remote peer‐mentorship programme including perceived support provided to the mentee, delivery challenges faced, and the potential impact of remote delivery on engagement.

Consenting participants completed an online consent form prior to being interviewed. All interviews were conducted by a female researcher experienced in qualitative interviewing (SM) who was not well known to the peer mentors. Interviews were conducted remotely via videoconferencing or telephone and lasted on average 70 min (range 41–109 min). Interviews were audio‐recorded and transcribed verbatim by an independent agency or Microsoft Teams/Zoom. Transcripts were checked for accuracy and anonymised by a researcher (SM or EL). Interviews focused on perception of training and support; the programme and resources; remote delivery of peer mentorship; and experience of being a peer mentor.

#### 2.7.3 Session Summaries

Peer mentors were asked to complete brief notes (session summaries) after each mentoring session to record which programme topics were covered, other topics discussed, and document their mentee's progress. Session summaries were collected by the study team at the end of the mentoring period.

The session summary forms were bespoke to this study. Completion of session summaries was covered in peer mentor training. Written instructions, examples and a template were provided for all peer mentors.

### 2.8 Data Analysis

Quantitative data from training evaluations and session summaries were analysed using descriptive statistics. Free‐text data were content‐analysed [30] by researchers (SM and EL). Meaning units were derived from the free text data and simple codes were applied. Working together, SM and EL developed and agreed on categories and combined these with quantitative data. The combined data were cross‐checked separately by researchers (SM and EL).

Reflexive thematic analysis [31, 32] was used to generate themes from peer mentor interview data. Two researchers (SM and EL) independently read and re‐read transcripts to familiarise themselves with the data. Transcripts were initially inductively coded and broadly categorised based on the research objectives of feasibility, acceptability, fidelity and perceived usefulness. Similarities and discrepancies were discussed, agreed and further explored with the wider study team. Codes and illustrative quotes were organised into categories using Microsoft Excel. Main themes relating to research objectives were developed. These themes were further examined with a focus on peer mentors’ experiences of delivering the intervention. Sub‐themes were developed and considered in relation to the training evaluation and session summary data.

## 3 Results

Twelve of fourteen peer mentors completed a training evaluation. Thirteen peer mentors delivered the peer mentorship programme. Twelve peer mentors consented and took part in remote interviews. One declined for personal reasons.

Twelve peer mentors returned summaries for each session delivered. One became uncontactable at the end of her mentoring and did not return session summaries. In total, summaries were returned for 123 of the 127 sessions delivered.

Findings from these three data sets were combined to form a narrative of the peer mentors’ perceptions and experiences of their role, including key programme delivery challenges. Findings focus on pre‐mentoring expectations and motivations to engage; and post‐mentoring reflections of their peer mentoring experience.

Three key themes were developed:

Theme 1: Aligning expectations of the peer mentoring role with mentoring experience.

Theme 2: Preparedness for peer mentoring.

Theme 3: Peer mentorship delivery challenges.

Quotes from qualitative interviews are labelled with peer mentors’ pseudonyms (without demographic details) to ensure that they are not identifiable.

## 4 Theme 1: Aligning Expectations of the Peer Mentoring Role With Mentoring Experience

Theme 1 was developed from interview data exploring peer mentors’ expectations of the role and post‐mentoring reflections on their overall experiences of mentoring. Session summaries were used to corroborate peer mentors’ reflections on attendance and provided insights into mentee's variable engagement with the programme.

### 4.1 Altruistic Motivation

Motivations and expressed expectations were broadly aligned, with most mentors stating that they joined the study because they wanted to make a positive difference to others and expected to do so through offering support and guidance. They wanted to share learning from their experience of living with OA, provide dedicated time for people to talk about OA, and encourage people to adopt helpful coping strategies. Some mentors recognised their altruistic motivation as a desire to *‘pay back’* the support they had received, and for *‘something good’* to come out of having OA.

[The programme] seemed really good and I thought it was, kind of, empowering if you had somebody who also had it, to talk to and go through it.

(PM‐Victoria)

### 4.2 Self‐Gain

Commonly mentors were motivated by a desire to interact with people in a similar position to themselves. They expected to learn about OA self‐management strategies and reinvigorate their own self‐management practice through interactions with mentees. One peer mentor expected her involvement to provide a focus for her own rehabilitation following recent hip surgery. A few mentors revealed that they had joined the study for financial gain and expected the volume of mentoring sessions to be greater than it was. As sessions were limited to six per mentee and with a total of 30 mentees in the study, this expectation of substantial financial gain was unrealistic.

### 4.3 Using Mentoring Skills

Peer mentors with previous mentoring experience expected to use skills such as motivational techniques, boundary setting and active listening. Those who were new to mentoring expected to learn and practice new skills. All peer mentors expected to support mentees practically by following the study programme and resources. Some mentors did not fully appreciate that mentoring would also involve emotional as well as practical support.

There was a lot more to it than what, you know, I thought. An awful lot more. It's just so much to take in and the hardest part is saying goodbye.

(PM‐Nora)

### 4.4 Engagement with the Programme

Peer mentors anticipated that mentees would be motivated to engage with the programme, attend all sessions and work to improve their OA self‐management. This expectation was not always met. Mentors reported that some mentees viewed mentorship as an opportunity to socialise or share their problems but were not ready to tackle the challenge of managing OA. Others reported that mentees who were already employing OA self‐management techniques such as exercising, pain management and eating healthily resisted trying new strategies such as activity pacing or goal setting. Mentors were empathetic to unengaged mentees but were frustrated by their lack of engagement with the programme.

I felt like I wasn't doing the study justice. I felt like I wasn't doing it properly. And I hate not doing things properly.

(PM‐Suzy)

Table 4 shows peer mentors’ expectations of mentoring with reflections on their experiences of mentoring in practice. This illustrates that for many mentors their expectations of the role were met. Mentors perceived that mentorship positively impacted on mentees; and both mentors and mentees gained from sharing knowledge and experience. However, in some cases these gains were associated with generalised peer support and not a consequence of programme delivery.

**Table 4:** Expectations and experiences of peer mentoring.

Expectation of peer mentoring	Examples of mentoring experience	Reflection on experience of peer mentoring in practice
To make a positive difference to others	Mentee reports on feeling more able to cope with OA.	*So it would be like a friendly thing. And I'd be working with somebody who's interested in improving their life as it stands …by finding ways to deal with their arthritis. And hopefully improve their arthritis because I've known through my own experience that exercises help me*. (PM‐Suzy)
	Mentor observes a change in mentee's mindset	*Instead of me, sort of being the driver she was actually sort of listening. She was actually telling me things rather than me having to ask*. (PM‐Patricia)
To benefit from being a peer mentor	Mentor develops mentoring skills	*I think I've got a lot out of it. I've got a lot out of how to communicate to people about just the different aspects of [OA] how they can lead their life. I think I've got a lot out of that*. (PM‐Isla)
	Mentor develops a sense of purpose	*Well, as a mentor, I felt I got as much from the mentees …as what hopefully I was giving to them. Because I was listening to their story and I realised, you know, there was a pattern to it*. (PM‐Victoria)
To connect with others and share lived experience of OA	Mentor develops sense of connections by sharing OA experience	*I just wanted to kind of interact with people who had similar experiences to me, where I could share my experiences of osteoarthritis with them*. (PM‐Behshad)
To gain satisfaction in the role	Mentee is motivated to engage with the programme	*They were both certainly motivated to do something, and I think this was because of their health, their physical health, not just because of their arthritis, but they had comorbidities. They both really wanted to do things. They both wanted to change*. (PM‐Richard)
	Mentor found mentoring interesting and purposeful	*I did really enjoy being part of her progress… for me to be able to do something that gave me a real sense of purpose, and was motivating for me too, was a really great thing to be part of*. (PM‐Lisa)
To learn about self‐managing OA	Mentor and mentee learn by sharing experiences	*We got to know each other better and it was a learning experience for me as well and I learned a lot about me in it, as well as helping them*. (PM‐Daphne)
		*[I] think it was useful for me. I think it was, hope it was, useful for them. So it was kinda like I mentioned, it was a two‐way interaction. So I think they perhaps learnt a few things from my experiences, and also stuff that you discuss from the booklets, the guides as well, and I think I picked up a few things from them as well*. (PM‐Behshad)
	Mentor and mentee learn through using programme resources	
To use mentoring skills	Mentor uses positive feedback to motivate mentee	*But the fact that she was engaged, and I think I was interested in her and gave her lots of positive feedback, I think that probably really helped our dynamic*. (PM‐Lisa)
	Mentor encourages two‐way interaction	*I would then dig a little deeper and ask what are you doing, rather than tell them [what to do] because I'm always very conscious that it should be a two‐ way conversation*. (PM‐Richard)
To take time to listen to mentee and offer support	Mentors tried to understand mentee in context of their lives	*Just time to look at different aspects of her life and how it all fits together and how her osteoarthritis management could improve as part of that. So, I think, I think the time factor was really important* (PM‐Lisa)
	Mentor takes time to listen to mentee as part of the mentoring process	*Both just needed to talk ‐ huge problems with chronic pain and other health issues and family stresses and everyday life. They needed someone with similar experience to talk to about dealing with OA on a daily basis*. (PM‐Nora)

## 5 Theme 2: Preparedness of Peer Mentors

Theme 2 was developed from pre‐mentoring training evaluations and post mentoring interview data to indicate how prepared and motivated peer mentors felt to deliver the OA self‐management programme.

### 5.1 Training Objective

Volunteers to the study naturally varied in their confidence and experience of mentoring in addition to their lived experience of OA. Training was designed to prepare volunteers to become effective peer mentors for the study i.e. deliver the self‐management programme as intended using programme resources and incorporating their experiences of self‐managing OA. Tailoring the programme to meet individual needs and preferences of mentees was highlighted as a key programme feature. However, preparing peer mentors beyond generalised tailoring was difficult given that mentees’ health and social circumstances were unknown when training was delivered.

### 5.2 Perceptions Immediately Post‐Training

Training evaluations completed immediately following training indicated that participants found the content interesting, informative and enjoyable. Participants praised the well‐planned presentations and liked the relaxed presentation style with multiple opportunities for discussion. Small group sessions made it easier for participants to interact and contribute.

Each part of the day was fully explained. Slides used were clear and easy to follow.

It was very informative and in‐depth for the time given.

People with greater prior knowledge of OA self‐management felt they did not gain much new information but rated the overall training experience positively.

Some participants found remote delivery tiring; others enjoyed the convenience of being in their home environment.

I would have preferred training to be in half day sessions, it was difficult to sit comfortably ‘til end of session.

Face to face would be nicer, easier to communicate.

A minority of participants raised concerns about their lack of mentoring experience and consequent suitability to offer support. This issue was highlighted by the role play scenarios involving PPI/past peer mentors. One participant called for training to include discussion of the disconnect between inexperience as a mentor and confidence to provide meaningful support to mentees.

Evaluations showed two‐thirds of participants felt ‘very equipped’ to fulfil the peer mentoring role. Out of a five‐point scale the median rating −5.0 (range 3–5). The majority (92%) also felt ‘4‐somewhat motivated’ or ‘5‐very motivated’ (median rating = 5.0, range 3–5) to carry out the role of peer mentor. Participants scored effectiveness of remote training as slightly lower, although 50% rated it as ‘5‐very effective’ (median rating = 4.5, range 3–5). Suggestions for improvement included shorter days, face to face delivery and more opportunities to practise peer mentoring through role play.

### 5.3 Perceptions Post‐Mentorship Delivery

In contrast, peer mentors provided mixed feedback on how well training prepared them for mentorship in their post‐mentoring interviews. Positive feedback included that they liked the structure of the training, valued the resources and felt more knowledgeable by the end. They appreciated the regular breaks and found interactive activities and discussions with past peer mentors most useful.

Several mentors reflected that training was difficult to fit around work or current lifestyle; others expressed a preference for shorter sessions citing OA and other health conditions as problematic. Some felt disconnected due to remote delivery, with technical connection difficulties and a perceived lack of group interaction impacting on their engagement.

I felt slightly removed from the experience of training. There were some really good speakers. It was quite intensive.

(PM‐Daphne)

Although peer mentors praised the comprehensive content, some felt, in retrospect, training had not always adequately prepared them to deliver the programme to study mentees. Unexpected aspects of mentoring included needing to pre‐plan sessions and generate examples from their own self‐management practices such as activity pacing and goal setting.

The delay between training and being matched with a mentee was problematic for several mentors as they felt they had forgotten aspects of their training and felt less well prepared to support mentees when the time came. Several peer mentors expressed a need for refresher sessions and opportunities to practise mentoring in the interim.

Most mentors indicated that training had given them the tools, motivation and confidence to deliver the programme. In retrospect, some felt unprepared for the wide variation in mentees’ attitude to self‐management; and underestimated the value of adapting their mentoring approach to meet the emotional needs of the mentee. They found supporting reticent and/or overwhelmed mentees particularly difficult.

## 6 Theme 3: Peer Mentorship Delivery Challenges

Theme 3 explores dominant mentoring delivery challenges predominantly using interview data, supported by sessions summaries.

Peer mentors identified challenges to delivering the mentorship programme. These were grouped into three interrelated challenge areas: attendance at sessions; delivering the self‐management programme as intended; and complex lives of mentees. Table 5 provides a breakdown of delivery challenges with examples and potential impact on peer mentors, the programme and mentees.

**Table 5:** Challenges of delivering the peer mentorship programme.

Sub‐theme	Challenge	Potential impact	Peer mentor quote
Attendance at sessions	Mentee attendance at session is low or irregular due to lack of mentee engagement	Incomplete self‐management programme	*But I think for [mentee] not to turn up, I just think he'd got a very dysfunctional life. I don't think he could organise himself…. He maybe didn't quite expect the commitment that was needed* (PM‐Richard)
		Peer mentor feels their support is de‐valued	*I think it made me more wishy‐washy waiting for [mentee] to turn up* (PM‐Suzy)
		Lower potential for behaviour change/peer support	*I think what disappointed me was when he said he hadn't even looked at the booklets he'd had. I felt really disappointed… I think he jumped in and maybe didn't realise what he was jumping into*. (PM‐Nora)
	Difficulty with interpersonal connection and/or technical connection	Lack of rapport between peer mentor and mentee	*Connecting face to face would feel a bit more real and may make developing rapport easier. It would be a little more real… it would feel a little more, just more immediate* (PM‐Mark)
		Peer mentor loses confidence in their ability to fulfil the role	*I got full to emotionally charged about it because you feel so ashamed if you don't know how to deal with technology, or at least I do, you know I just feel ashamed* (PM‐Daphne)
Delivering the programme as intended	Mentee perceives that programme topics are not relevant to them	Mentorship session is used to explore non‐programme topics	*I think one of the problems is because of the workbook, because you were very restrained, so what I was finding mainly with most people that they had already started to do some of those things*. (PM‐Richard)
		Peer mentor adapts session to keep mentees engaged	*I felt a responsibility with it, you know because I didn't want them to suddenly just give up in the middle of it. I wanted to get them to the end so I had to keep it light‐hearted, you know just keep it going so the next week they would want to come back*. (PM‐Victoria)
	Mentee is resistant to following programme	Peer mentor becomes frustrated and/or loses confidence to provide mentorship support	*It scared me a bit. I'm thinking oh my god what can I say that she's actually going to agree with? Or how can I put things where she doesn't see them as impossible?* (PM‐Patricia)
	Key OA self‐management topics e.g. goal setting, healthy eating, exercise are not covered	Mentee less likely to benefit from improved self‐management behaviour	*The hardest [topic] I found to deliver for most of them was the goal setting. I think people don't, in general, people don't like to be pinned down and they don't want to make promises they're not going to keep*. (PM‐Suzy)
			*So she just said she ate healthily, her sister did it, but she ate too much. So she knew all about how to eat proper food and everything but she said her sister got very angry with her if she didn't eat all her food*. (PM‐Victoria)
			*We just didn't get to [exercise] I think doing the exercise sessions would have…I don't think she would've found value in that and I don't think she would've engaged with that*. (PM‐Patricia)
Complex lives of mentees	Competing demands on mentee e.g. caring responsibilities, work commitments, medical appointments	Mentee unable to focus on mentorship session.	*I thought right I've got to do an hour with this lady and it just was not going to happen. You could just see, on the [screen] that she couldn't sit still. She'd, as I say, you know, go off on a tangent, and yeah, it just, and it kind of put me off a little bit, kind of threw me a little bit. I didn't expect that*. (PM‐Isla)
		Mentee de‐prioritises OA self‐management	*I just felt that I was mentoring somebody who had such a lot on his plate*. (PM‐Daphne)
	Mentee has multiple long‐term conditions in addition to OA	Peer mentor loses confidence to deliver the programme	*When you are dealing with supporting people with comorbidities it can be very difficult. You cannot necessarily empathise with everything or even be knowledgeable in some of those conditions to an extent that you are really helpful* (PM‐Melanie)
		Mentee resistance to engage with key topics such as exercise	*And she was really adamant every time, so I didn't want to push it. She did have her own medical things going on*. (PM‐Lisa)
	Session attendance and/or engagement with programme is irregular due to fatigue and poor health	Mentee does not complete the programme	*I think she just didn't understand the relevance of it. And possibly just didn't have the time or the energy. You know, if you're heavily medicated up to the eyes on painkillers and whatever, which they both were, not just for their arthritis, but other quite serious health conditions. You know what I mean. It's hard, isn't it*. (PM‐Isla)

### 6.1 Attendance at Sessions

One unanticipated challenge to delivery was low and irregular attendance of mentees at sessions. Less than half of mentees (43%) attended all 6 sessions within an 8‐week period. Although 17% of mentees attended 4 or 5 sessions, a large percentage (40%) only attended 3 sessions or fewer. Common reasons for low attendance amongst mentees were low engagement with the programme, lack of rapport with their peer mentor or not feeling able to commit to regular weekly 1‐hour sessions.

Poor attendance meant peer mentors were unable to progress through the programme core topics in the allocated time resulting in some feeling disappointed or questioning their competence to support the mentee.

When low mentee attendance was perceived as lack of commitment to the programme, this often negatively impacted on development of a supportive mentor‐mentee relationship. It also had practical implications for some mentors, leaving them feeling frustrated and disrespected.

I don't get enough sleep at night, whether I have a nap or not. So having a bit of extra sleep is really helpful for me and committing to a time window and then having people rudely just don't bother turning up and don't even let you know.

(PM‐Suzy)

Peer mentors who recognised lack of commitment or time for mentees to attend sessions, adopted a more ambivalent approach to mentoring, perhaps preparing less for the session, or recognising that mentees still benefited from shorter more social sessions.

In some cases, peer mentors understood that low attendance was due to competing demands or other health issues preventing attendance, and although sympathetic to the mentee, felt exasperated by unexpected disruption to the mentoring sessions.

However, some peer mentors were discouraged by their mentee's reluctance to identify self‐management goals and lack of perceived ‘progress’.

It was a bit difficult to engage with her…. I had to basically prompt her and poke, almost poke and prod her to get things out of her.

(PM‐Emma)

Session summaries indicated that mentees attending fewer sessions did not appear to adopt key self‐management practices and covered fewer core topics.

### 6.2 Delivering the Programme As Intended

Peer mentors reported experiencing challenges to delivering the programme as intended. These included inconsistent attendance and variable engagement of mentees as illustrated by quotes above; low confidence of peer mentors to tailor programme delivery to meet mentees’ needs; and ability of peer mentors to modify their mentoring approach.

The peer mentor role was to provide information, skills and motivation to enable mentees to identify goals (e.g. activity, dietary) and to support mentees to progress towards their goals. Some mentees appeared reluctant to identify self‐management goals, others were so overwhelmed by the impact of OA on their lives, (persistent pain, fatigue and restricted mobility) that practising self‐management techniques did not appear possible. They simply needed to talk to someone with similar experience about dealing with OA in the context of their lives.

I was trying to follow a set process, and he just really wanted to listen to me talk about what was going on and what he was doing. And that helped, that was helping him.

(PM‐Nora)

Some mentors felt constrained by the programme resources and although they tried to follow them faithfully, became aware that some key topics were not sufficiently comprehensive to be useful to their mentee. This resulted in mentees losing interest in the programme and peer mentors feeling unable to offer meaningful support. In response, some modified the programme by expanding on key topics, exploring barriers to change or introducing new topics of interest to the mentee such as how to apply for financial benefits or home adaptations.

Peer mentors sometimes experienced difficulty with groups of mentees’ where social interactions dominated the session. The challenge of balancing mentee engagement with adherence to the programme left some peer mentors unable to offer constructive support. However, one mentor found that listening to the group chat, enabled her to identify topics of interest and suggest potential goals for the mentees.

Several peer mentors reported feeling restricted by remote programme delivery. Despite muscle‐strengthening exercise being an integral part of the programme, not all mentors covered this topic and only a few practised exercises alongside their mentee in sessions.

I was very unsure about doing the exercises remotely, I've got to be honest. And they were a bit of a challenge, I've got to be honest.

(PM‐Victoria)

Mentors felt that discussing exercise and exploring resources was feasible via telephone/videoconferencing but were reluctant to guide their mentee through exercises remotely, suggesting safety and lack of space as reasons.

Covered the included home activity plan section in the handouts with exercises, going through the safety checklist, and then the exercise themselves. [Mentee] did not want/expect me to physically demonstrate the exercises, which was difficult to do on Zoom, and instead we went through them together, one by one to understand what each entailed and how they are undertaken.

(PM‐Richard, Session Summary week 3)

Mentors who judged that mentees would be resistant to exercising in sessions, often chose not to introduce the topic. However, others reported positive results from mentees who followed the exercises in the resource pack.

Sheila feels activity helps and is trying to integrate more exercise into her daily life. She keeps the pack in view and open at the exercise pages to remind her to work through the exercises that she can manage (lunges and sideways leg raises are too hard). When watching TV she gets up and moves during the commercial breaks.

(PM‐Daphne, Session Summary week 2)

### 6.3 Complex Lives and Competing Demands of Mentees

As highlighted previously, people experiencing socioeconomic disadvantage are more likely to have MLTCs. The baseline data collected with our mentees prior to being matched indicated that almost all (90%) had other health conditions alongside hip or knee OA, accompanied by debilitating symptoms such as pain, fatigue, lack of mobility, depression or anxiety. Several mentees who suffered from sleep disturbance struggled to attend morning sessions or concentrate for the duration of the session. Despite regular reminders, some simply forgot to attend.

Many mentees cared for family members, or had work or family commitments such as attending appointments, running errands, working overtime, which they prioritised over their own needs. This may have led to them de‐prioritising OA self‐management and not engaging well with the programme.

Peer mentors reported being apprehensive about delivering some programme topics to mentees with multiple health conditions, for example, encouraging physical activity with mentees with cardiovascular or respiratory disease; or discussing healthy eating sensitively with overweight mentees. Some also felt ill‐prepared to offer OA guidance to mentees with low mood, where their mental health concerns overshadowed the physical health concerns. Peer mentors who focused on building rapport with their mentee and encouraging a sense of empowerment coped well in these circumstances.

Complex lives, competing demands and de‐prioritising OA self‐management whilst managing other health conditions meant some mentees disengaged with the programme, struggled to follow their peer mentor's guidance and some mentees withdrew from the study early. Faced with mentoring under such challenging circumstances, a few peer mentors also chose to finish mentoring after being matched with only one mentee.

## 7 Discussion

This paper provides novel insights into the expectations and experiences of volunteer peer mentors delivering an OA self‐management programme to people experiencing socioeconomic disadvantage from the perspective of the peer mentors. Peer mentors were integral to the self‐management programme and therefore offered unique perspectives on the intervention. The findings provide practical insights into how peer mentoring programmes can be designed to meet peer mentors’ expectations; ensure peer mentors are well prepared for, and appropriately supported in, their role; and address challenges to successful mentorship programme delivery.

Exploring peer mentors’ expectations demonstrated that mentors wanted to provide value and be valued by others through offering self‐management support and fulfilling their expectation to deliver the programme. Learning about OA self‐management and connecting with others with OA were also key motivators. Mentors’ experiences of delivering the mentorship programme appeared to shift their initial pre‐mentoring altruistic motivation towards a gain‐orientated desire to receive positive feedback, develop rewarding relationships, and observe signs of behaviour change in their mentee.

Similarly, mentors’ perspectives of training pre‐ and post‐delivery of the mentorship programme revealed a shift in perception of preparedness. Peer mentors rated their specialist training positively but realised that it had not sufficiently prepared them for some of the challenges associated with mentoring people experiencing socioeconomic disadvantage and/or MLTCs. We identified three interrelated challenges which impacted on peer mentors’ motivation, confidence and ability to deliver the self‐management programme. Overcoming the challenges relied on peer mentors’ adapting their mentoring approach to match mentees’ individual needs. Some were able to do this through developing rapport and listening to mentees’ primary concerns, providing a therapeutic foundation on which OA self‐management could be developed. Other peer mentors persisted with an approach that was less well aligned to their mentees’ needs and/or lost confidence in their mentorship skills.

The key challenges identified in this study predominantly originated from mentees’ circumstances and readiness to engage with the programme. Mentees were self‐selecting volunteers to the programme and as such we anticipated that they would be ready to embrace OA self‐management. While this was true for some mentees, others with complex health and social needs may have been seeking less specific support from their mentor. Whilst they benefitted from peer mentorship, they were reluctant to engage with the self‐management programme. In some cases, this created a dilemma for mentors who wanted to both support their mentee and fulfil their (and our) expectation to deliver the self‐management programme.

Peer support literature highlights that ‘successful’ interventions are built on collaborative therapeutic relationships [14, 33] with peers who develop reciprocal trusting alliances [18, 34]. Interventions that fail to consider how mentees perceive their health condition(s) in relation to their everyday life may be unhelpful [35]. For example, older adults with MLTCs may prioritise current quality of life over perceived negative impact of lifestyle change such as diet and physical activity [36]. Additionally, culture‐specific and socioeconomically sensitive peer support may facilitate self‐management of long‐term conditions [37]. In this study, mentors who took time to understand how OA is perceived by mentees in the context of their lives were better able to adapt their approach and provide more beneficial support.

This peer mentorship programme was developed through adapting our previous in‐person peer mentorship programme [26]. This study's findings build on learning gained during the adaptation process. For example, training was delivered over two non‐consecutive days, with more breaks, to help reduce fatigue and maintain interest. Findings suggest this approach was partly successful but could be improved further by offering shorter sessions and in‐person training options if feasible.

These findings also build on a qualitative evaluation of our previous in‐person peer mentorship programme [29]. As in this study, the peer mentors who delivered the in‐person programme were motivated by a desire to help others and were largely positive about the training, albeit they found training had not fully prepared them for their role. Conversely, there were notable differences in the peer mentors’ experiences of delivering the mentorship programme. Mentee attendance with the previous in‐person peer mentorship programme was high, contrasting with the low and irregular attendance in this study. Mentees’ other health conditions appeared to be an important contributor to low/irregular attendance and limited engagement with self‐management in this study. Delivering mentorship programmes that focus on self‐management of MTLCs rather than a single condition could potentially help address this and maximise impact. The remote format of this intervention may also have made it easier for mentees to simply not turn up to mentorship sessions, compared to mentors visiting mentees in‐person in their own home or workplace.

Attendance issues in this study negatively impacted peer mentors, causing frustration and loss of confidence in the role. Wider literature emphasises that people delivering peer support need to feel supported and valued [33]. In this study, perceiving a positive change in mentee's behaviour or wellbeing because of peer mentorship was highly motivating. Sometimes this was evident within the mentorship period although often mentors were left unsure of their impact on mentees until they received post‐mentoring feedback by the study team. This study's peer mentors received ongoing support from the study team with regular check‐ins and updates, however, informal group support from other peer mentors may have helped them feel more connected to the study and to each other. In a real‐world environment, having an online forum or mentoring buddy would benefit mentors who, for example, want to recap on training, discuss dealing with reluctant mentees or managing groups.

## 8 Strengths and Limitations

The main strength of this study was the significant involvement of people with OA and experience of socioeconomic disadvantage (PPI and peer mentors) in the development and delivery of the peer mentorship programme. Harnessing their experience and paying attention to their views enabled us to understand the impacts of peer mentorship on mentees and mentors. Learning from the challenges they faced will guide future programme development.

The peer mentors were diverse in key characteristics (Table 3), although the ethnic diversity of peer mentors (77% White British) was lower than the ethnic diversity of mentees. Limited time for recruitment is a possible contributing factor to this.

Matching of mentors and mentees was controlled by availability and time constraints of the study. Although personal compatibility was a strong consideration for matching, some dyads were matched predominately on availability rather than anticipated compatibility. This may have made developing rapport more challenging. Having a larger pool of peer mentors and running the study over a longer period would improve capacity for matching, although this does not guarantee improved mentor‐mentee rapport.

This was the first time all the peer mentors had delivered this programme and so were all inexperienced in this specific programme. How effectively the peer mentors engaged with their mentees may have impacted how engaged the mentees were with the programme and suggested activities and self‐management strategies.

There was no direct assessment of how well the peer mentors delivered their mentoring as this would have required a member of the research team to be present during mentorship sessions, and/or recording of sessions. Presence of research staff has potential to affect rapport between mentors and mentees and may result in reluctance to openly share their lived experience with one‐another.

Another limitation was that the training evaluation survey and session summary template were bespoke to this study. This ensured that they were tailored to the needs of the study and mentorship programme but were not tested for validity and reliability. However, using these multiple connected sources of information provides rich data on how this remote peer mentorship programme functions from the perspectives of the people delivering it.

## 9 Implications for Practice and Future Research

This study's findings provide practical insights into points to consider when designing peer mentorship programmes for practice and future research. Table 6 provides design considerations in relation to the expectations and challenges faced by peer mentors. Overall, the findings emphasise the need to set realistic expectations with peer mentors and mentees before they commit to the programme; maximise flexibility in both the peer mentor training and mentorship programme delivery; and ensure peer mentors receive ongoing support as well as comprehensive initial training.

**Table 6:** Summary of suggestions for peer mentorship programme design.

Expectations, learning and challenges		Suggestions for peer mentorship programme design
Peer mentors’ expectations of peer mentoring	To make a positive difference to others	Regularly ask mentees for feedback and ensure positive and constructive feedback is passed onto mentors.
	To benefit from being a peer mentor	Ask mentors what skills they would like to develop and aim to incorporate those into the initial training and ongoing mentor development activities. Support peer mentors to reflect on the learning they gain from the training and mentorship delivery. Ensure expectations about payments are clearly understood by peer mentors before they commit to the programme.
	To connect with others and share lived experience of OA	Ensure training highlights the mutual benefits of peer mentoring and building good mentor‐mentee relationships. Offer discussion forums for peer mentors, so they can share their experiences of OA as well as mentoring with each other.
	To gain satisfaction in the role	Encourage peer mentors to share feedback on how they are finding the role and where possible use their feedback to address challenges and promote satisfaction in the role.
	To learn about self‐managing OA	Ensure the initial training, resources, and any ongoing development activities promote learning about self‐management. Offer discussion forums for peer mentors, so they can share their experiences of self‐management.
	To use mentoring skills	Offer more opportunities for role play in initial and ongoing training to allow peer mentors to practise using their mentoring skills, ideally using real‐world examples from previous mentorship programme delivery. Support mentors to reflect on how they are using their mentoring skills in practice and how they could develop and use their skills further.
	To take time to listen to mentee and offer support	Ensure mentorship programmes are designed to be flexible and include sufficient time for peer mentors to listen to their mentees. Include setting OA in the context of mentees’ lives in training.
Learning from peer mentors’ experiences of training and mentoring	Training should prepare peer mentors to meet mentees’ individual needs and preferences	Provide training on how to meet mentees’ differing individual needs and preferences, including their emotional needs, using real‐world examples where possible. Include training session on adapting mentoring approach to meet mentee's needs.
	Training should build peer mentors’ confidence to deliver mentorship.	Ensure training addresses needs of inexperienced and experienced mentors and builds confidence to provide meaningful support to mentees. Provide ongoing support to help build mentors’ confidence in the role, for example, offer weekly online forum with past peer mentors to tackle challenges experienced during mentoring.
	Training should address how to pre‐plan sessions.	Deliver training on how to pre‐plan mentorship sessions and generate examples from the peer mentor's own experiences of using self‐management practices such as activity pacing and goal setting. Offer one to one sessions with past peer mentors to help with planning sessions and introducing sensitive topics.
	Training should be friendly and interactive.	Deliver training using a friendly and relaxed style with opportunities for discussion, small group sessions, and role play sessions.
	Training with past peer mentors is valuable.	Involve past or current peer mentors in delivering training and providing ongoing support.
	Remote training may be preferred by some peer mentors but not others.	Consider offering in‐person and remote training options. Provide technical assistance for remote training.
	Training may be tiring.	Ensure training sessions include regular breaks. Consider delivering training in half‐day rather than full‐day sessions.
	Training may be difficult for peer mentors to fit around other commitments.	Consider offering training at flexible times, such as daytime and evening sessions.
	Delays between training and being matched with a mentee may lead to peer mentors forgetting aspects of training and feeling unprepared.	Aim to deliver training shortly before matching peer mentors with mentees and offer refresher sessions and ongoing opportunities to practise mentoring.
Challenges faced by peer mentors	Low or irregular attendance at sessions by mentees	Ensure training prepares peer mentors for the possibility of low and irregular attendance. Ensure clear expectations around attendance are set with mentees and mentors before they commit to the programme. Address barriers to low or irregular attendance, such as offering the intervention in different formats and on a rolling basis. Support flexibility in the scheduling of mentorship sessions where possible.
	Difficulty with interpersonal connection and/or technical connection	Include interpersonal skills training and offer support to mentors as necessary to help develop good interpersonal connections. Recruit a large and diverse pool of peer mentors to help optimise peer mentor and mentee matching. Enable peer mentors and mentees to be rematched if difficulties with a particular interpersonal connection cannot be addressed. Offer flexibility with the format of programme delivery, such as Zoom, WhatsApp or telephone and address any technical connection challenges.
	Mentee perceives that programme topics are not relevant to them	Promote flexibility in mentorship programmes so mentees can choose topics that are relevant to them. Involve past peer mentors in sessions – with mentor's and mentee's agreement – to help guide mentees towards core and optional topics.
	Mentee is resistant to following programme	Ensure clear expectations about the mentorship programme content are set with mentees before they commit to the programme. Support mentors to explain the benefits of following the self‐management programme to mentees and how to encourage them to follow it. Consider providing mentees with a forum to share their experiences of being on the programme.
	Key OA self‐management topics e.g. goal setting, pacing, exercise are not covered	Provide mentors with training and support in how to explain the importance of the core topics. Address concerns related to specific core topics, such as difficulty practising exercises remotely. Consider offering separate group exercise sessions alongside the mentoring programme.
	Mentee lack of engagement with the programme	Ensure clear expectations around engagement are set with mentees before they commit to the programme. Support mentees to attend sessions by sharing success stories based on previous mentees. Ensure training prepares peer mentors for the possibility of low engagement with the programme and how to adapt mentoring approach.
	Irregular attendance at sessions and engagement with self‐management due to lack of time, poor health fatigue or medical appointments	Ensure clear expectations around time commitments are set with mentees before they commit to the programme. Offer shorter mentorship sessions if needed to help address fatigue challenges. Offer flexibility with the timing of mentorship sessions.
	De‐prioritising OA self‐management	Support peer mentors to understand OA self‐management in the context of MLTCs. Offer guidance on re‐focusing sessions to incorporate OA and other long‐term conditions. Consider delivering mentorship programmes that focus on self‐management of MTLCs rather than a single condition.

## 10 Conclusion

Peer mentorship is a potentially valuable approach to supporting people to self‐manage long‐term conditions such as OA. However, there are inherent challenges of providing support for, and with, people with OA experiencing socioeconomic disadvantage, including consistent availability and prioritising long‐term benefit over current need. This study's findings offer practical insights for designing peer mentoring programmes that align with mentors’ expectations, provide adequate preparation and support, and address challenges to effective delivery. Setting realistic expectations with peer mentors and mentees could help promote attendance, engagement and satisfaction. Better preparing and supporting peer mentors for flexible delivery and broadening the programme approach from condition specific management to MLTC management is also likely to improve applicability, appeal and impact.

## Author Contributions

**Elizabeth Lavender:** conceptualisation, investigation, funding acquisition, writing – original draft, methodology, writing – review and editing, formal analysis, project administration. **Samantha Mason:** investigation, methodology, formal analysis, writing – original draft, writing – review and editing. **Anna M. Anderson:** conceptualisation, funding acquisition, formal analysis, investigation, methodology, writing – original draft, writing – review and editing. **Linda Eckersley:** conceptualisation, funding acquisition, methodology, writing – review and editing. **Susan Barry:** conceptualisation, funding acquisition, methodology, writing – review and editing. **Gretl A. McHugh:** conceptualisation, funding acquisition, writing – original draft, methodology, supervision, writing – review and editing, formal analysis.

## Ethics Statement

Ethical approval for this study was gained from the South Birmingham Research Ethics Committee (23/WM/0108). All participants provided informed consent prior to participating. The project was performed in accordance with all relevant guidelines and regulations, including the Declaration of Helsinki. All peer mentors and mentees provided informed consent prior to participating in the study. Peer mentors also provided written consent to be interviewed and for their pseudonymised quotes to be used for study purposes.

## Conflicts of Interest

The authors declare no conflicts of interest.

## Supporting information

Supporting File

## Data Availability

The data that support the findings of this study are available on request from the corresponding author principal investigator/corresponding author – Professor Gretl McHugh (G.A.McHugh@leeds.ac.uk). The data are not publicly available due to privacy or ethical restrictions.
