# Supplementary material for: Expectations and Experiences of Peer Mentors in Supporting People With Osteoarthritis and Socioeconomic Disadvantage: A Multi‐Method Exploration of Challenges and Lessons Learned
Source: Health Expect. 2026 Aug 16;29(4):e70819. doi: 10.1111/hex.70819 (PMC13477603; doi:10.1111/hex.70819)
Supplement: Supplementary file 1 [file HEX-29-e70819-s001.docx]

**Supplementary File 1: Consolidated criteria for reporting qualitative studies (COREQ): 32-item checklist**

| **No. Item** | **Guide questions/description** | **Reported on Page #** |
| --- | --- | --- |
| **Domain 1: Research team and reﬂexivity** | | |
| *Personal Characteristics* | | |
| 1. Interviewer/facilitator | Which author/s conducted the interview or focus group? | 7 |
| 2. Credentials | What were the researcher’s credentials? E.g. PhD, MD | Available via ORCID profile |
| 3. Occupation | What was their occupation at the time of the study? | 7 |
| 4. Gender | Was the researcher male or female? | 7 |
| 5. Experience and training | What experience or training did the researcher have? | 7 |
| *Relationship with participants* | | |
| 6. Relationship established | Was a relationship established prior to study commencement? | 4 - 6 |
| 7. Participant knowledge of the interviewer | What did the participants know about the researcher? e.g. personal goals, reasons for doing the research | 7  Interviewer was a member of the study team not known well by most peer mentors |
| 8. Interviewer characteristics | What characteristics were reported about the inter viewer/facilitator? e.g. Bias, assumptions, reasons and interests in the research topic | Not reported due to manuscript word limit |
| **Domain 2: study design** | | |
| *Theoretical framework* | | |
| 9. Methodological orientation and Theory | What methodological orientation was stated to underpin the study? e.g. grounded theory, discourse analysis, ethnography, phenomenology, content analysis | 7-8  Main analytical approach was reflexive thematic analysis |
| *Participant selection* | | |
| 10. Sampling | How were participants selected? e.g. purposive, convenience, consecutive, snowball | 4 |
| 11. Method of approach | How were participants approached? e.g. face-to-face, telephone, mail, email | 4 |
| 12. Sample size | How many participants were in the study? | 4, 7 |
| 13. Non-participation | How many people refused to participate or dropped out? Reasons? | 8 |
| *Setting* | | |
| 14. Setting of data collection | Where was the data collected? e.g. home, clinic, workplace | 7 |
| 15. Presence of non-participants | Was anyone else present besides the participants and researchers? | Unknown as interviews were conducted remotely |
| 16. Description of sample | What are the important characteristics of the sample? e.g. demographic data, date | 6, table 3 |
| *Data collection* | | |
| 17. Interview guide | Were questions, prompts, guides provided by the authors? Was it pilot tested? | 7 |
| 18. Repeat interviews | Were repeat interviews carried out? If yes, how many? | Abstract, 7 |
| 19. Audio/visual recording | Did the research use audio or visual recording to collect the data? | 7 |
| 20. Field notes | Were ﬁeld notes made during and/or after the interview or focus group? | Not reported due to manuscript word limit |
| 21. Duration | What was the duration of the inter views or focus group? | 7 |
| 22. Data saturation | Was data saturation discussed? | N/A as all peer mentors were invited to participate in a qualitative interview. |
| 23. Transcripts returned | Were transcripts returned to participants for comment and/or correction? | Transcripts not returned due to time constrictions |
| **Domain 3: analysis and ﬁndings** | | |
| *Data analysis* | | |
| 24. Number of data coders | How many data coders coded the data? | 7-8 |
| 25. Description of the coding tree | Did authors provide a description of the coding tree? | Not reported due to manuscript word limit |
| 26. Derivation of themes | Were themes identiﬁed in advance or derived from the data? | 8 |
| 27. Software | What software, if applicable, was used to manage the data? | MS Excel |
| 28. Participant checking | Did participants provide feedback on the ﬁndings? | PPI members provided feedback on the findings. Not reported due to manuscript word limit |
| *Reporting* | | |
| 29. Quotations presented | Were participant quotations presented to illustrate the themes/ﬁndings? Was each quotation identiﬁed? e.g. participant number | 8 - 14, tables 4 & 5 |
| 30. Data and ﬁndings consistent | Was there consistency between the data presented and the ﬁndings? | 8 - 15, tables 3 – 5, figure 1 |
| 31. Clarity of major themes | Were major themes clearly presented in the ﬁndings? | 8 - 15 |
| 32. Clarity of minor themes | Is there a description of diverse cases or discussion of minor themes? | 8- 15 |

**Supplementary File 2: Volunteer Peer Mentor Role Description**

**Remote Osteoarthritis peer-mentorship (RaMIgO) Study**

**Volunteer Position:** Volunteer Peer Mentor

**Reports to:** Study Coordinator

**Study background**

Osteoarthritis is a common joint condition mainly affecting older people. Hip and

knee osteoarthritis may cause high levels of pain, social isolation and difficulty with

daily activities. Osteoarthritis is more common in people experiencing

socioeconomic disadvantage, and its impact is more severe in this group. Health

inequalities in osteoarthritis are fuelled by socioeconomic factors such as poorer

general health, reduced access to/uptake of health services and digital exclusion.

Alternative and better forms of support are needed to support people with

osteoarthritis who are experiencing socioeconomic disadvantage, a group often

underserved by research.

Self-management helps people with osteoarthritis to continue to live well. For

example, lifestyle changes can reduce the day-to-day impact of the condition by

reducing pain and improving function and mood. Our previous research developed

and evaluated a programme that involved training volunteers with osteoarthritis to

become ‘peer mentors’ to provide self-management support to others with the

condition. Limitations of this research were that most study participants were older,

white, well-educated females and the programme was only delivered face-to-face.

Wider evidence suggests that the impact of self-management programmes may

vary between different groups of people. A tailored, remote peer support

intervention would be valuable for people experiencing socioeconomic

disadvantage who are often underserved by health research, therefore increasing

existing health inequalities.

**Overview of the role**

The RaMIgO study aims to develop and evaluate a remote peer support model

(volunteer peer mentor) to improve self-management of individuals with OA.

Volunteer peer mentors will provide peer-mentorship remotely (either by telephone

or video-call) on a weekly basis for a period of around 6 weeks, for around 1 hour

per week. Our working definition of a peer mentor is that he/she is usually

someone who has a specific condition and has undergone training to be able to

provide focused assistance and mentorship to someone with the same condition.

The RaMIgO project will train a small group of volunteers to become peer mentors.

As a peer mentor your skills and personality will be matched appropriately to an

individual living with osteoarthritis of the hip or knee.

**Criteria**

The criteria for this role are that you:

- are living with osteoarthritis of the hip or knee;
- feel that you are disadvantaged because of your finances, education or social circumstances;
- have time to carry out the role;
- agree to maintain confidentiality.

**Key requirements of the role**

- Undertake an enhanced disclosure (DBS) check;
- Attend (either remotely or face-to-face) RaMIgO training on [Insert Dates];
- Be available to volunteer for a minimum of 1 hour per week for at least 6 weeks, ideally being involved for a longer period of 2-5 months;
- Participate in the evaluation of the study (this will involve recording a brief summary about your peer-mentorship sessions);
- Participate in an interview about your experience of being a Peer Mentor (if you wish to);
- Inform the Study Coordinator of any concerns about the participant(s) you are providing peer-mentorship to.

**Key skills of the role**

- Be a good listener
- Be able to use your own experience to help others
- Build a sensitive, respectful and caring relationship with study participants
- Be interested in helping people to find ways to solve their problems
- Be understanding rather than judgemental about a person’s situation or circumstances
- Respect the confidentiality of participants at all times
- Establish and maintain appropriate boundaries with participants

**What will you gain from this role?**

- You will be helpful in supporting individuals to manage their osteoarthritis;
- You will gain training and support in self-management of osteoarthritis as a long-term condition;
- You will be able to meet new people and use your skills to help them;
- You will have the chance to find out more about services and activities in your local area.

**Payments and support**

You will be able to claim a £20 recognition (thank you) payment for each session

you deliver. The recognition payment may affect your financial situation, such as

affecting any benefits you receive. You will be free to decline the payments or ask

for a smaller amount if you prefer. However, declining payment can still have

benefit implications.

**Benefits**

At times, accepting a payment can affect people’s state benefits. If you are

receiving benefits, and are concerned about this, then you can find more

information here: <https://www.nihr.ac.uk/documents/payment-guidance-for-members-of-the-public-considering-involvement-in-research/27372>.

**Tax**

If you are paid via the IR35 non-exempt form, the payment will go straight into your

bank account. The University of Leeds will not take tax and National Insurance off

at source. It’s your responsibility to declare income to HMRC, if necessary. You can

find out more information here: <https://www.gov.uk/check-if-you-need-tax-return>.

Unfortunately, we are not able to offer individual advice about benefits or tax, as

people's circumstances differ so much. However, we are happy to provide a letter

describing the nature of your involvement and why it is different to employment.

**Other expenses**

As well as offering a recognition payment, we include any out-of-pocket expenses

and additional support with Wi-Fi. We can also cover expenses related to

childcare, respite, personal support or communication needs.

**Extra support**

We will arrange any extra support you need to carry out this role, such as providing

a training manual and help with digital access. We will provide regular

supervision/support meetings/phone-calls with the Study Coordinator for support

and to exchange information or discuss any concerns regarding participants. We

can provide additional support with translation or interpreting.

**Data protection**

We will need to store basic personal details about you if you carry out this role.

These will include your name, email address and the financial details we need to

pay you. We will store all your personal details on the University of Leeds secure

servers in line with the University data protection procedures.

Only people employed by the University who are directly involved in the study /

payment process will have access to your personal details, and your details will

only be used when essential. More details about the University of Leeds data

protection procedures are available at: <https://dataprotection.leeds.ac.uk/>.

**Research team members [ xxxxxx ]**

**Contact details [xxxxxxx]**

If you would like to discuss this role further, please contact [name], via [email]or

phone [study mobile]

***Thank you for considering becoming a Volunteer Peer Mentor for this study***

This project has been funded by the Nuffield Foundation’s Oliver Bird Fund and

Versus Arthritis (now Arthritis UK) (OBF/FR-000023819), but the views expressed

are those of the authors and not necessarily the funders. Visit

[www.nuffieldfoundation.org](http://www.nuffieldfoundation.org) and [www.versusarthritis.org](http://www.versusarthritis.org) (now: <https://www.arthritis-uk.org>)

**Supplementary File 3: Figure 1 image description**

Flow chart of the process for the intervention delivery, showing the delivery steps and accompanying information. An arrow demonstrates that ongoing peer mentor support from the study team was provided throughout the process.

The steps involved in the intervention delivery include:

- Mentee matched with peer mentor.
- Programme resources sent to mentees.
- Arrange and schedule remote introductory meeting between peer mentor and mentee (one to one) or arrange and schedule remote introductory meeting between peer mentor and mentees (group).
- Session one, including facilitated introduction taking place on week one, followed by sessions two to six, with session six taking place on week six to eight.

The accompanying information includes:

- Peer mentor is a trained volunteer with hip and/or knee OA experiencing socioeconomic disadvantage.
- Peer mentor completes session summaries after each session to document notes and topics covered with mentee(s).
- Study team assists with scheduling of peer mentoring sessions, sets up and starts video call (if used) and sends reminders of session to mentee(s) and peer mentor.
- Sessions cover core and optional topics.
- Core topics: Learning about OA; Getting Active; Activity pacing; Goal setting; Eating well; Managing pain.
- Optional topics: Getting motivated; Relaxing and sleeping; Communicating well; Having a joint replaced; Getting support for work; Getting connected.
